# Dedicated Pediatricians in Emergency Department: Shorter Waiting Times and Lower Costs

**DOI:** 10.1371/journal.pone.0161149

**Published:** 2016-08-26

**Authors:** Manuel Rocha Melo, Manuel Ferreira-Magalhães, Filipa Flor-Lima, Mariana Rodrigues, Milton Severo, Luis Almeida-Santos, Alberto Caldas-Afonso, Pedro Pita Barros, António Ferreira

**Affiliations:** 1 Department of Pediatrics, Integrated Pediatric Hospital, Centro Hospitalar de S. João, Porto, Portugal; 2 Institute of Public Health, University of Porto, Porto, Portugal; 3 Center for Health Technologies and Information Systems Research, Faculty of Medicine, University of Porto, Porto, Portugal; 4 Department of Clinical Epidemiology, Predictive Medicine and Public Health, Faculty of Medicine, University of Porto, Porto, Portugal; 5 Lisbon Nova University, Lisboa, Portugal; 6 Centre for Economic Policy Research, London, United Kingdom; 7 Centro Hospitalar de S. João, Porto, Portugal; Old Dominion University, UNITED STATES

## Abstract

**Background:**

Dedicated pediatricians in emergency departments (EDs) may be beneficial, though no previous studies have assessed the related costs and benefits/harms. We aimed to evaluate the net benefits and costs of dedicated emergency pediatricians in a pediatric ED.

**Methods:**

Cost-consequences analysis of visits to a pediatric ED of a tertiary hospital. Two pediatric ED Medical Teams (MT) were compared: MT-A (May–September 2012), with general pediatrics physicians only; and MT-B (May–September 2013), with emergency dedicated pediatricians. The main outcomes analyzed were relevant clinical outcomes, patient throughput time and costs.

**Results:**

We included 8,694 children in MT-A and 9,417 in MT-B. Medication use in the ED increased from 42.3% of the children in MT-A to 49.6% in MT-B; diagnostic tests decreased from 24.2% in MT-A to 14.3% in MT-B. Hospitalization increased from 1.3% in MT-A to 3.0% in MT-B; however, there was no significant difference in diagnosis-related group relative weight of hospitalized children in MT-A and MT-B (MT-A, 0.979; MT-B, 1.075). No differences were observed in ED readmissions or in patients leaving without being seen by a physician. The patient throughput time was significantly shorter in MT-B, with faster times to first medical observation. Within the cost domains analyzed, the total expenditures per children observed in the ED were 16% lower in MT-B: 37.87 euros in MT-A; 31.97 euros in MT-B.

**Conclusion:**

The presence of dedicated emergency pediatricians in a pediatric ED was associated with significantly lower waiting times in the ED, reduced costs, and similar clinical outcomes.

## Introduction

There are different models of pediatric emergency care worldwide.[1–5] Differences in facilities, age of admission, triage, referral or physician’s qualification may influence the outcomes in pediatric emergency care.[6–8]

Over the last decade, pediatric emergency medicine has been progressively recognized, [9–10] first in the United States, Canada, [11, 12] and Australia and later in Europe, [13, 14] where it is now regarded as a subspecialty by the European Academy of Pediatrics.[15] However, the practice still varies widely across different countries and regions, [4] and little is known about the benefits of having dedicated emergency pediatricians in a pediatric ED.

Specific performance measures for pediatric emergency care were identified and should be used to assess efficiency of pediatric emergency care systems.[16, 17] Evidence-based guidelines are required to standardize pediatric emergency care models and clinical practice procedures. In this context, analysis of the net benefits and related costs of dedicated emergency pediatricians teams are of major importance. Cost-consequences analysis is a comprehensive economic evaluation that fits the society’s health values, [18] and can be applied to the assessment of dedicated emergency pediatricians in ED. Objective results of not only costs, but also consequences, will inform decision-makers and improve emergency care for children.

The present study aimed to assess the net benefits and costs of dedicated emergency pediatricians in a pediatric ED. The specific aims were to analyse the following outcomes: clinical outcomes; patient throughput time; and costs of hospital medication, diagnostic tests, consumable materials, and medical staff.

## Patients and Methods

### Study Setting

Portuguese National Health System is a Beveridge-based system, supported by taxes and, therefore, the access is universal and free for every citizen.

Centro Hospitalar de São João (CHSJ) is a public, mixed adult-children, and university tertiary-level hospital located in Porto, Portugal. Pediatric ED occupies a separate place within the hospital facilities, the catchment area is approximately 700 000 children/adolescents (0–17 years-old, inclusive) and there are on average 80 000 visits per year. Patients may direct themselves to the ED or be referred by a health professional, and the admissions to this ED are universal and totally free of charge. The patients undergo triage by trained nurses in accordance with the Pediatric Canadian Triage and Acuity Scale (Paed CTAS).[19]

Medical staffing working in this ED is organized in 8 medical teams composed by physicians from three different hospitals: four teams from CHSJ, three from Centro Hospitalar do Porto, and one from Matosinhos Hospital. Each team ensures coverage of 24 hours per week, divided into two shifts of 12 hours.

Until 2013, each shift comprised a medical team with five ‘general pediatrics consultants’–MT-A. In May 2013, in accordance with national guidelines, [20] CHSJ reorganized the medical staff for its four pediatric medical teams. After this reorganization, three ‘pediatric consultants dedicated to the ED’ and two ‘general pediatrics consultants’ composed each shift—MT-B. The medical teams were the only aspect that changed between these two periods; every other aspect of pediatric ED remained the same: facilities, electronic medical systems, non-medical staff (nurses and auxiliary personnel), and triage procedures.

‘General pediatricians’ were consultant physicians with board certification in pediatrics. These general pediatricians work 40 hours per week in inpatient, outpatient clinics and ED; each of these consultants work 12 hours per week in the pediatric ED. ‘Pediatricians dedicated to the ED’ were also consultant physicians with board certification in pediatrics; however, these consultants had an additional multidisciplinary training in pediatric emergency medicine. This training was conducted in full-time regimen (40 hours per week) over a 6-month period, and included practice in: pediatric intensive care, neonatal intensive care, pediatric interhospital transport, orthopedics, otorhinolaryngology, general surgery, and anesthetics. After this training period, pediatricians dedicated to ED started to work 40 hours per week exclusively in pediatric ED and interhospital transport of critically ill children.

### Study Design and Participants

We performed a cost-consequences analysis of the implementation of MT-B in our pediatric ED, using patients’ electronic medical records and hospital administrative databases. To examine the performance of MT-A and MT-B, we selected comparable annual periods in consecutive years: May 1 to September 30, 2012 for MT-A; and May 1 to September 30, 2013 for MT-B.

We included all patients (0–17 years-old, inclusive) that visited the pediatric ED of CHSJ. The exclusion criteria were: patients evaluated by pediatric medical teams from Centro Hospitalar do Porto and Matosinhos Hospital; and patients referred directly to other specialties in triage. Patients who left the pediatric ED without being seen by a physician (dropouts) were excluded from the overall analysis, but the frequency was compared between the two periods.

Confidentiality of participants was assured by patient records anonymization and de-identification prior to analysis. This study was reviewed and approved by the Ethics Committee of CHSJ, Porto, Portugal.

### Outcome Measures

The main comparisons were relevant clinical outcomes, patient throughput times, and costs. We also analysed demographic characteristics. Discharge diagnoses were classified in accordance with the International Classification of Diseases, Ninth Revision (ICD-9).[21]

The relevant clinical outcomes considered were: need for medication within the ED; need for diagnostic tests within the ED; number of hospitalisations; readmission to ED in the 72-hour period after discharge; death in the ED; and patients leaving without being seen by a physician. In order to address potential differences in hospitalisations, we assessed the complexity of the hospitalised patients with the diagnosis-related group relative weight.[22]

Patient throughput time was defined as the time from patient arrival to time of discharge from pediatric ED. This time was further divided into the following durations between: arrival and triage; triage and first medical observation; and first medical observation and discharge.

Cost analysis included four different cost domains: hospital medication given inside the ED; diagnostic tests; clinical consumables; and medical staff (regular salary and overtime remuneration).

As stated before, besides differences in medical teams composition, no other change occurred between MT-A and MT-B; also, our analysis was focused in costs potentially influenced by medical staff management. So we did not account for the costs with nursing, security professionals, facilities, administrative consumables or other general costs.

### Statistical Analysis

Categorical variables (clinical outcomes) were characterized by counts and proportions. Numerical variables (throughput times) that presented a right-skewed distribution were log transformed and described with geometric mean (mean_g_) and standard deviation (SD_g_). To compare numerical variables after log transformation, we used two-independent sample *t* test; for categorical variables, we employed a chi-square test. We categorized the effect size of the comparisons between the two ED models according to Cohen.[23] Considering that we are working with large samples, measures of effect size were preferred over significance tests to remove the dependence on sample size and the associated high probability of significant differences. For continuous variables (or the respective log transformation) the effect size was calculated as the difference between two means (MT-A and MT-B) divided by the pooled standard deviation. For categorical variables we used the formula $\sqrt{\sum \begin{array}{l} i \end{array}{\left(p_{1i}-p_{0i}\right)}^{2}/p_{0i}}$, where *p*_0i_ is the proportion of the *i*^th^ cell under *H*_0_ and *p*_1i_ is the proportion of the *i*^th^ cell under *H*_1_ (observed proportion). For comparisons of the categorical variables (clinical outcomes), the effect size of 0.1–0.3 was considered small, 0.3–0.5 medium, and >0.5 large. For comparisons of numerical variables (throughput times), the effect size of 0.2–0.5 was considered small, 0.5–0.8 medium, and >0.8 large.

To measure the association between clinical outcomes and the main exposure, we used the odds ratio (OR) and respective 95% confidence interval (95%CI). We estimated the OR by means of an unconditional logistic regression.

We performed the cost analysis using the total cost in each time period (sum of the four cost domains for MT-A and for MT-B) divided by the number of patients observed in each one. The results for the costs are presented in euros per patient observed in the pediatric ED. We also performed a multiple quantile regression for the costs of diagnostic tests since that was the only cost domain where all the costs were specifically allocated to each patient observed in the ED.[24] We compare the diagnostic tests costs in MT-B with MT-A adjusting for age, month of admission, and level of triage. In particular, a quantile regression model follows the changes in coefficients and can indicate heterogeneity in the direction and magnitude of the associations between predictor variable (MT-B vs. MT-A) and the linear dependent variable (costs of diagnostic tests) from the mean estimates.

We conducted the statistical analysis using SPSS version 22 (SPSS IBM, New York, NY, USA). Quantile regression was estimated using the library quantreg from the software R 2.14.1.[25] The level of significance was fixed at 0.05.

## Results

**Fig 1:**
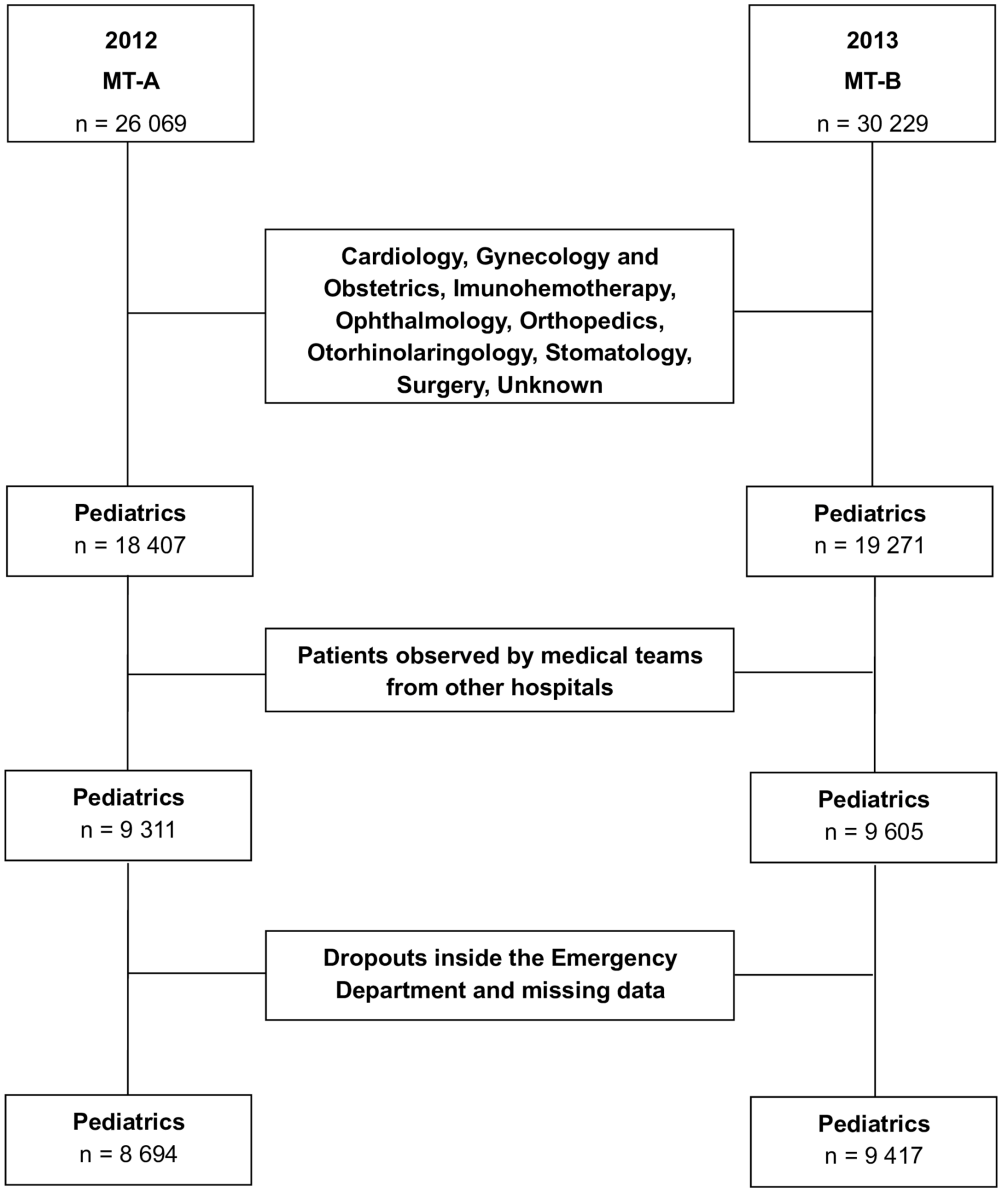
Study flowchart. Patients included in each medical team (MT-A and MT-B) are shown; exclusion criteria appear in the central boxes.

From a total of 56 298 visits to pediatric ED, we included 8 694 (15.4%) in MT-A and 9 417 (16.7%) in MT-B. The study flowchart is presented in Fig 1 and demographic data summarized in Table 1. In the final sample, children aged 1 to 5 years old were the most frequent patients visiting the pediatric ED (MT-A—n = 4 282, 49.3%; MT-B—n = 4 666, 49.5%).

**Table 1:** Sample characteristics.

	Total	MT-A	MT-B	p value
	(n = 18 111)	(n = 8 694)	(n = 9 417)	
**Gender, n (%)**				
** Male**	9 402 (51.9)	4 494 (51.7)	4 908 (52.1)	0.565
** Female**	8 709 (48.1)	4 200 (48.3)	4 509 (47.9)	
**Age, n (%)**				
** <12 months**	2 306 (12.7)	1 004 (11.5)	1 302 (13.8)	
** 12–24 months**	2 836 (15.7)	1 367 (15.7)	1 469 (15.6)	
** 1–5 years**	6 112 (33.7)	2 915 (33.5)	3 197 (33.9)	<0.001
** 6–10 years**	3 306 (18.3)	1 654 (19.0)	1 652 (17.5)	
** 11–15 years**	2 316 (12.8)	1 147 (13.2)	1 169 (12.4)	
** ≥16 years**	1 235 (6.8)	607 (7.0)	628 (6.7)	
**Month of visit, n (%)**				
** May**	3 906 (21.6)	2 099 (24.1)	1 807 (19.2)	
** June**	3 829 (21.1)	1 537 (17.7)	2 292 (24.3)	
** July**	3 560 (19.7)	1 913 (22.0)	1 647 (17.5)	<0.001
** August**	3 086 (17.0)	1 485 (17.1)	1 601 (17.0)	
** September**	3 730 (20.6)	1 660 (19.1)	2 070 (22.0)	
**Origin, n (%)**				
** Primary care**	818 (4.5)	412 (4,7)	406 (4.3)	
** Other hospital**	153 (0.9)	82 (0.9)	71 (0.8)	
** Private clinic**	105 (0.6)	53 (0.6)	52 (0.6)	0.115
** Health-care call center**	402 (2.2)	172 (2.0)	230 (2.4)	
** Outpatient department**	22 (0.1)	9 (0.1)	13 (0.1)	
** Without referral**	16 611 (91.7)	7 966 (91.7)	8 645 (91.8)	
**Canadian triage level, n (%)**				
** Level 1 and 2**	954 (5.3)	426 (4.9)	528 (5.6)	
** Level 3**	8 455 (46.7)	4 117 (47.4)	4 338 (46.1)	
** Level 4**	8 027 (44.3)	3 845 (44.2)	4 182 (44.4)	0.049
** Level 5**	675 (3.7)	306 (3.5)	369 (3.9)	
**Triage destination, n (%)**				
** Pediatric resuscitation room**	161 (0.9)	55 (0.6)	106 (1.1)	
** Observation ward**	629 (3.5)	211 (2.4)	418 (4.4)	<0.001
** Waiting room**	17 321 (95.6)	8 428 (97.0)	8 893 (94.5)	
Disorders according to ICD-9*, n (%)				
** Infectious**	2 869 (16.0)	1 456 (16.9)	1 413 (15.1)	
** CNS and Sense organs**	1 480 (8.2)	692 (8.0)	788 (8.4)	
** Respiratory system**	3 477 (19.3)	1 460 (16.9)	2 017 (21.6)	
** Digestive system**	1 226 (6.8)	613 (7.1)	613 (6.6)	0.213
** Signs and symptoms**	4 180 (23.2)	2 034 (23.6)	2 146 (22.9)	
** Injury and poisoning**	2 062 (11.5)	975 (11.3)	1 087 (11.6)	
** Others**	2 696 (15.0)	1 402 (16.2)	1 294 (13.8)	

ICD-9: International Classification of Diseases, Ninth Revision; CNS: Central Nervous System.

Paed CTAS level 3 patients were the most common (MT-A—n = 4 117, 47.4%; MT-B—n = 4 338, 46.1%). MT-B had more level 1 and level 2 Paed CTAS episodes than MT-A (MT-A—n = 426, 4.9%; MT-B—n = 528, 5.6%; *p* = 0.049), as well as more resuscitation room (MT-A—n = 55, 0.6%; MT-B—n = 106, 1.1%; *p*<0.001) and observation ward admissions (MT-A—n = 211, 2.4%; MT-B—n = 418, 4.4%; *p*<0.001). There were no statistically significant differences regarding gender and the origin of children that visited pediatric ED.

### Clinical Outcomes

The number of children that received medication increased from 42.3% in MT-A to 49.6% in MT-B. Children who underwent diagnostic tests decreased from 24.2% in MT-A to 14.3% in MT-B (Table 2). The number of children hospitalized increased from 1.3% in MT-A to 3.0% in MT-B. However, all the effect sizes of these comparisons were extremely low (Table 2). There was no significant difference on the diagnosis-related group relative weight of hospitalized children (MT-A, 0.979; MT-B, 1.075; p = 0.45).

**Table 2:** Clinical outcomes: proportions, effect size, and odds ratio (crude and adjusted) for comparisons between the two medical teams (MT-A and MT-B).

	Total, n (%)	MT-A, n (%)	MT-B, n (%)	Effect size	Crude OR (95%CI)	Adjusted OR (95%CI)
**Medication use**	8 348	**3 677**	**4 671**	0.103	**1.34**	**1.40**
	(46.1)	**(42.3)**	**(49.6)**		**(1.27–1.42)**	**(1.31–1.49)**
**Diagnostic test use**	3 453	**2 106**	**1 347**	0.178	**0.52**	**0.52**
	(19.1)	**(24.2)**	**(14.3)**		**(0.48–0.56)**	**(0.49–0.57)**
**Hospitalization**	397	**115**	**282**	0.082	**2.30**	**2.26**
	(2.2)	**(1.3)**	**(3.0)**		**(1.85–2.87)**	**(1.81–2.83)**
**Readmission**	1 264	575	689	0.020	1.12	1.11
	(7.0)	(6.6)	(7.3)		(0.99–1.25)	(0.99–1.25)

Bold values: *p*<0.001 for comparisons between the MT-A and MT-B

OR, odds ratio; CI, confidence interval

Adjusted OR: adjusted to age, month of visit, and level of triage.

There was no statistically significant difference regarding readmissions to pediatric ED in the 72-hour period after discharge. In the periods under study, there was only one death within the ED (MT-A). There were 73 (0.8%) patients who left without being seen by a physician in MT-A and 85 (0.9%) in MT-B (*p* = 0.652).

### Patient throughput time

Patient throughput time in the pediatric ED was significantly shorter in MT-B, as well as all the subdivisions of this time (Table 3). The effect sizes of those differences were classified as small except for duration between patient arrival and first medical observation, which had a medium effect size.

**Table 3:** Patient throughput time (hours) in pediatric emergency department—comparison between the two medical teams (MT-A and MT-B).

	Total, mean_g_ (SD_g_)	MT-A, mean_g_ (SD_g_)	MT-B, mean_g_ (SD_g_)	Effect size
**Patient throughput time in ED (hours)**	1.84 (2.47)	**2.08 (2.43)**	**1.65 (2.47)**	0.248
** Duration between arrival and triage (hours)**	0.12 (1.98)	**0.13 (2.06)**	**0.11 (1.88)**	0.220
** Duration between triage and first medical observation (hours)**	0.28 (3.79)	**0.39 (3.32)**	**0.20 (3.91)**	0.513
** Duration between first medical observation and discharge (hours)**	0.48 (2.41)	**0.58 (2.41)**	**0.40 (2.33)**	0.414

Bold values: *p*<0.001 for comparisons between MT-A and MT-B.

SD_g_, geometric standard deviation.

The mean_g_ duration between arrival and triage was less than 10 minutes in both MT-A and MT-B. The mean_g_ duration between triage and first medical observation was 23.4 minutes in MT-A and 12 minutes in MT-B (p<0.001; effect size 0.513) and was inversely proportional to the priority levels of triage (S1 Fig).

### Cost Analysis

**Fig 2:**
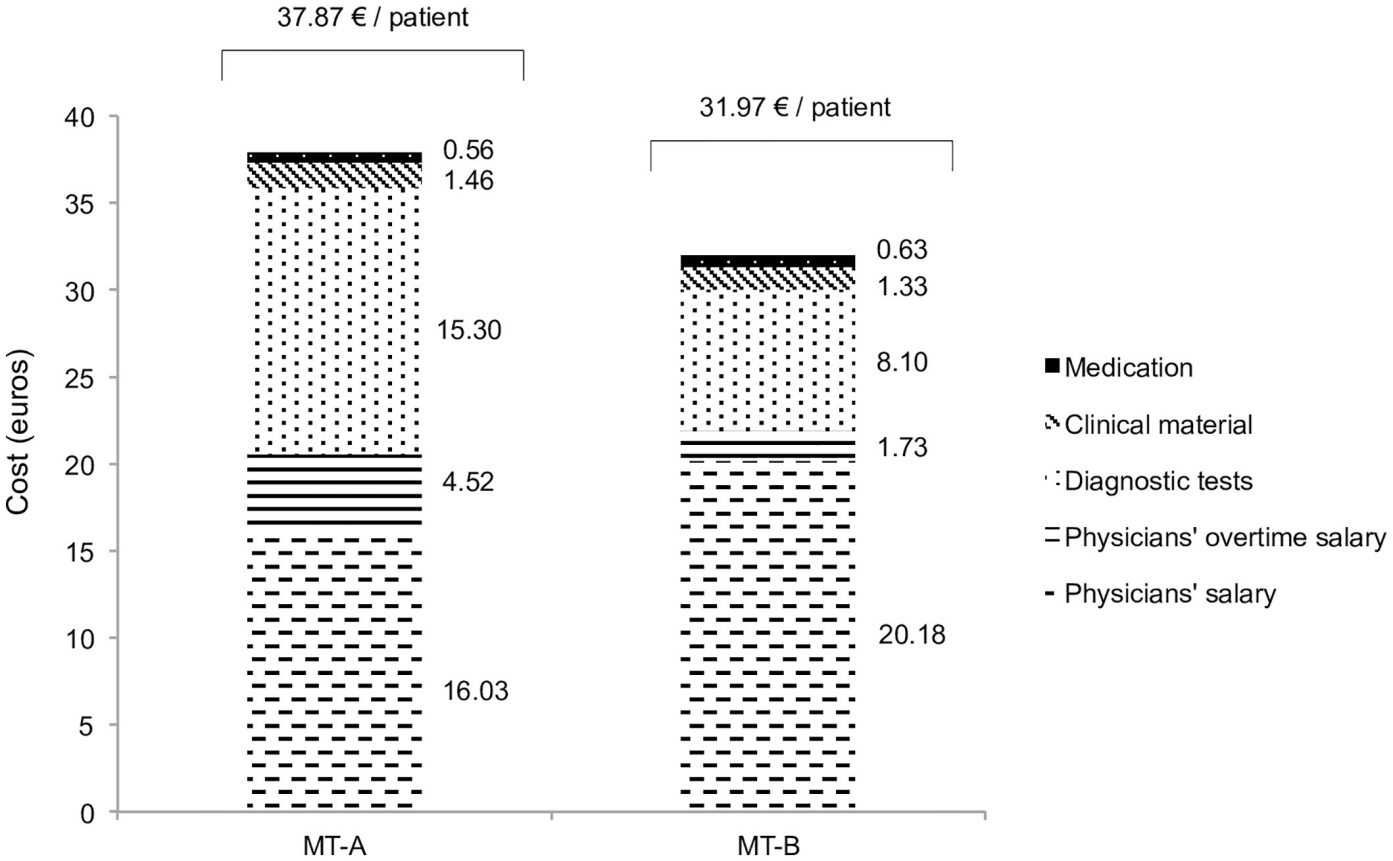
Specific costs per patient observed in the pediatric emergency department in medical teams (MT-A and MT-B).

Within the cost domains analysed, the total expenditure per patient observed in the pediatric ED was 16% lower in MT-B (37.87 euros in MT-A and 31.97 euros in MT-B; Fig 2). The relative costs of diagnostic tests, clinical consumables, and physicians’ overtime pay were lower in MT-B (relative decrease of 47%, 9%, and 62%, respectively). The relative costs of medication and physicians’ regular salary were higher in MT-B (relative increase of 26% and 13%, respectively).

In the multivariate quantile regression, we observed that the highest savings in diagnostic tests observed in MT-B were mainly driven by a lower use of the cheaper (38% lower) and the costliest (15% higher) diagnostic tests. The costs for medium expenditure with diagnostic tests were similar in the two models (S2 Fig).

## Discussion

This is the first study that evaluates net benefits (clinical outcomes and patient throughput time) and costs of dedicated emergency medicine pediatricians in the pediatric ED. The cost-consequences approach allowed us to perform a comprehensive analysis, aiming to impact the future decision-making in pediatric emergency care. We were able to describe and compare the two models using most of the recommended performance indicators for pediatric ED with the highest utility score, as ranked by the Royal College of Physicians and Surgeons of Canada.[16]

In our study, the differences between general pediatricians and pediatricians dedicated to ED were mainly two: 1) the training program, i.e. a 6-month extra training period in pediatric emergency medicine for pediatricians dedicated to ED; 2) clinical activity, i.e. pediatricians dedicated to ED have a full-time work (40 hours per week) in pediatric ED and interhospital transport of critically ill children, as opposed to general pediatricians that work 40 hours per week in several areas of pediatric department (inpatient, outpatient clinics and ED) and, of these, only 12 hours per week in ED. We propose that the differences in training and clinical practice may give a greater know-how in treating children in ED, leading to better outcomes in the emergency setting: higher confidence in managing medication in the ED and less diagnostic tests use may decrease waiting times in the ED and, altogether, decrease the costs in pediatric ED.

### Clinical Outcomes

There is a lack of published studies about diagnostic tests and medication prescription within the pediatric ED. In our study, we observed a decrease in diagnostic testing in MT-B and an increase in medication use, even when adjusted for patient age, level of triage, and month of ED visit. According to the specificity of the training program and clinical activities, pediatricians dedicated to ED were probably more prone to have an integrated approach to the children admitted to ED. This may explain why pediatric emergency physicians have had more restrictive criteria for employing diagnostic testing in an emergency setting and, in turn, may have had greater confidence in managing medication. Further studies are needed to address this hypothesis. Nevertheless, those differences were considered clinically irrelevant (see the low effect size in Table 2) and may also be related to our large sample size and the power to detect small differences. This point also applies to the increase in hospitalisations observed in MT-B (see the low effect size in Table 2). Moreover, we did not observed any difference in diagnosis-related group relative weight between the two periods, indicating that physician’s clinical criteria on 14ospitalization remained the same in MT-A and MT-B. The overall rate of 14ospitalization observed is similar to that found in other pediatric ED settings.[26]

The rates of readmission to the ED after 72 hours and of patients left without being seen were extremely low in both periods and no differences were found. Previous studies have reported higher proportions (7%-16%) of patients leaving without being seen.[27,28]

### Patient throughput time

The total patient throughput time inside the pediatric ED, as well as all the subdivided times, were significantly shorter in MT-B, with acceptable effect sizes (small to medium effect sizes; Table 3). We believe that this reduction in duration may be partly a consequence of the fewer diagnostic tests performed and lack of need to wait for test results.

These findings support the effectiveness of dedicated emergency pediatricians in reducing waiting times / length of stay in the pediatric ED. Keijzers et al. reported a reduction of the total ED length of stay (in an adult-child mixed ED) with a pediatric medical team, but they were unable to find an effect on the waiting time to see a doctor.[29] In our setting, both models were composed of pediatricians only (or pediatric residents); the difference was in ‘general pediatricians’ vs ‘dedicated emergency pediatricians’.

We did not make satisfaction inquiries, however, previous reports have demonstrated the importance of shorter waiting times on carer satisfaction levels and in the likelihood of recommending the pediatric ED.[29]

### Costs

MT-B showed a 16% reduction in the total costs analysed (medications, diagnostic tests, clinical consumables, and physicians’ salaries). The highest saving was in physicians’ overtime pay (62% reduction). That saving was accompanied by a slight increase in the physicians’ regular salaries. That change was anticipated since the emergency pediatricians had been hired, and consequently general pediatrics physicians worked less overtime.

The cost domain that most affected the final cost reduction in MT-B was diagnostic tests. The 47% decrease amounted to a reduction of 7.2 euros per patient observed. The savings were greater among patients who underwent less expensive diagnostic tests (38% lower) and in those who received the most costly diagnostic tests (15% higher), meaning that fewer cheaper and costly diagnostic tests were performed (the number of diagnostic tests of medium prices remained similar).

### Strengths

Portuguese National Health System access is universal and free for every citizen so, our study population is representative of all the children, without having the possible confounding effect of social factors like health insurance or socioeconomic status. MT-A and MT-B differed only in the composition of the pediatric medical teams; all the other features were identical. This allowed us to assess the effect of having dedicated emergency pediatricians in a pediatric ED. In addition, we focus our analysis on the children attended by pediatricians and excluded patients referred to other specialties (Fig 1). We also chose the same time of year in 2012 and 2013 to minimize possible bias related to the incidence of acute pathologies linked to different seasons. Finally, the study sample size allowed us to greatly reduce the probability of type II errors.

### Limitations

This study had some limitations. There were small differences in some baseline characteristics between the two models (Table 1); however, we adjusted our estimates to those variables, and the differences in the baseline characteristics were probably detected because of the large sample. The comparison between two different periods may had also not account for the small changes in practices that could have been occurred; nevertheless, we did not identify any major change in practices in pediatric ED. Finally, our results are fitted to the population of the studied hospital, and extrapolation to other settings has to be made with caution.

## Conclusion

The presence of dedicated emergency pediatricians in a pediatric ED was associated to significantly lower waiting times in the ED, less diagnostic tests use and reduced costs. Clinical outcomes were similar in the two models studied. More studies on utility, benefits and costs of specialized pediatric emergency teams in pediatric ED are needed in order to help decision makers to improve pediatric emergency care models, maximizing health results in this setting.

## Supporting Information

S1 Fig Comparison of estimated marginal means of duration between triage and first medical observation in MT-A and MT-B. Blue line: MT-A. Green line: MT-B.
(TIFF)

S2 Fig Multivariate quantile regression for costs with diagnostic tests in MT-B compared with MT-A, adjusted for age, month of admission, and level of triage. Slash-dotted line: mean difference of MT-B compared with MT-A. Gray zone: 95% confidence interval for the mean difference. Red solid line shows the mean difference of MT-B compared with MT-A using the usual least squares linear regression.
(TIFF)

## References

[1] Van Veen M, Moll HA. Reliability and validity of triage systems in paediatric emergency care. *Scand J Trauma Resusc Emerg Med*. 2009;17:38 10.1186/1757-7241-17-38 19712467 PMC2747834

[2] Fernández A, Pijoan JI, Ares MI, Mintegi S, Benito FJ. Canadian Paediatric Triage and Acuity Scale: assessment in a European paediatric emergency department. *Emergencias*. 2010;22:355–360.

[3] American Academy of Pediatrics, Committee on Pediatric Emergency Medicine; American College of Emergency Physicians, Pediatric Committee; Emergency Nurses Association Pediatric Committee. Joint policy statement—guidelines for care of children in the emergency department. *Pediatrics*. 2009;124(4):1233–1243. 10.1542/peds.2009-1807 19770172

[4] Mintegi S, Shavit I, Benito J, REPEM group (Research in European Paediatric Emergency Medicine). Paediatric emergency care in europe: a descriptive survey of 53 tertiary medical centers. *Pediatr Emerg Care*. 2008;24(6):359–363. 10.1097/PEC.0b013e318177a762 18562877

[5] Models of Emergency Care. Sydney. NSW Ministry of Health; 2012. http://www.health.nsw.gov.au/Performance/Publications/ed-model-of-care-2012.pdf Accessed January 29, 2016.

[6] Sacchetti A, Baren J, Carraccio C. The paradox of the nested pediatric emergency department. *Acad Emerg Med*. 2005;12(12):1236–1239. 16293893 10.1197/j.aem.2005.06.029

[7] Davies F, Gaushe-Hill M, Chu S, Cheema B, Ang A, Caceres L, et al. 2012 International Standards of Care for Children in Emergency Departments. Melbourne. International Federation for Emergency Medicine—Paediatric Special Interest Group; 2012. http://www.ifem.cc/Resources/PoliciesandGuidelines.aspx Accessed September 24, 2015.

[8] Dharmar M, Marcin JP, Romano PS, Andrada ER, Overly F, Valente JH, et al Quality of Care of Children in the Emergency Department: Association with Hospital Setting and Physician Training. *J Pediatr*. 2008;153(6):783–789. 10.1016/j.jpeds.2008.05.025 18617191 PMC9724612

[9] Li M, Baker MD, Ropp LJ. Pediatric emergency medicine: a developing subspecialty. *Pediatrics*. 1989;84(2):336–42. 2748264

[10] Pou Fernández J, Benito Fernández J. Pediatría de urgencias: una nueva especialidad. *An Esp Pediatr*. 2002;56(1):2–4. 11792261

[11] Curriculum Subcommittee, Section of Emergency Medicine, American Academy of Pediatrics. Pediatric emergency medicine (PEM) fellowship curriculum statement. *Pediatr Emerg Care*. 1993;9(1):60–66. 8488151 10.1097/00006565-199302000-00019

[12] Babl FE, Weiner DL, Bhanji F, Davies F, Berry K, Barnett P. Advanced training in pediatric emergency medicine in the United States, Canada, United Kingdom, and Australia: An international comparison and resources guide. *Ann Emerg Med*. 2005;45(3):269–275. 15726049 10.1016/j.annemergmed.2004.10.003

[13] Benito J, Mintegi S, Ruddy RM, Gonzalez del Rey JA. Changing Clinical Practices and Education in Pediatric Emergency Medicine Through Global Health Partnerships. *Clin Pediatr Emerg Med*. 2012;13(1):37–43.

[14] Salter R, Maconochie IK. Implementation of recommendations for the care of children in UK emergency departments: national postal questionnaire survey. *BMJ*. 2005;330(7482):73–74. 15579478 10.1136/bmj.38313.580324.F7 PMC543867

[15] Benito J, Mintegi S, Luaces C, Liviana D, Chérón G, Martinot A, et al (Working Group on Paediatric Emergency Medicine). The European Syllabus for Paediatric Emergency Medicine. European Academy of Paediatrics; Paediatric Section of the UEMS; UEMS Section of the European Board of Emergency Medicine; 2011. http://www.simeup.com/doc/syllabus.pdf Accessed January 29, 2016.

[16] Hung GR, Chalut D. A consensus-established set of important indicators of pediatric emergency department performance. *Pediatr Emerg Care*. 2008;24(1):9–15. 10.1097/pec.0b013e31815f39a5 18165798

[17] Alessandrini E, Varadarajan K, Alpern ER, Gorelick MH, Shaw K, Ruddy RM, et al Emergency department quality: An analysis of existing pediatric measures. *Acad Emerg Med*. 2011;18(5):519–526. 10.1111/j.1553-2712.2011.01057.x 21569170

[18] Coast J. Is economic evaluation in touch with society's health values? *BMJ*. 2004;329(7476):1233–6. 15550430 10.1136/bmj.329.7476.1233 PMC529373

[19] Gouin S, Gravel J, Amre DK, Bergeron S. Evaluation of the Paediatric Canadian Triage and Acuity Scale in a paediatric ED. *Am J Emerg Med*. 2005;23(3):243–247. 15915392 10.1016/j.ajem.2004.02.046

[20] Paiva JA, Silva AM, Almeida AL, Seco CM, Gomes CM, Ribeiro E, et al. Reavaliação da Rede Nacional de Emergência e Urgência; 2012. http://www.portaldasaude.pt/NR/rdonlyres/0323CC90-45A4-40E4-AA7A-7ACBC8BF6C75/0/ReavaliacaoRedeNacionalEmergenciaUrgancia.pdf Accessed January 29, 2016.

[21] International Classification of Diseases, Ninth Revision (ICD-9). World Health Organization; 2011. http://www.cdc.gov/nchs/icd/icd9cm.htm Accessed January 29, 2016.

[22] Fetter RB, Shin Y, Freeman JL, Averill RF, Thompson JD. Case mix definition by diagnosis related groups. *Med Care*. 1980;18(2):1–53. 7188781

[23] Cohen J. A power primer. *Psychol Bull*. 1992;112(1):155–159. 19565683 10.1037//0033-2909.112.1.155

[24] Koenker RW, Bassett QW. Regression quantiles. *Econometrica*. 1978;46:33–50

[25] R Core Team (2013). R: A language and environment for statistical computing. R Foundation for Statistical Computing, Vienna, Austria. http://www.R-project.org/. Accessed January 29, 2016.

[26] Hampers LC, Faries SG, Poole SR. Regional after-hours urgent care provided by a tertiary children’s hospital. *Pediatrics*. 2002;110(6):1117–1124. 12456908 10.1542/peds.110.6.1117

[27] Ng Y, Lewena S. Leaving the paediatric emergency department without being seen: understanding the patient and the risks. *J Paediatr Child Health*. 2012;48(1):10–15. 10.1111/j.1440-1754.2011.02187.x 21988657

[28] Gaucher N, Bailey B, Gravel J. Who Are the Children Leaving the Emergency Department Without Being Seen by a Physician? *Acad Emerg Med*. 2011;18(2):152–157. 10.1111/j.1553-2712.2010.00989.x 21314774

[29] Keijzers G, Crilly J, Walters B, Crawford R, Bell C. Does a dedicated pediatric team within a busy mixed emergency department make a difference in waiting times, satisfaction, and care transition? *Pediatr Emerg Care*. 2010;26(4):274–280. 20401972 10.1097/pec.0b013e3181d6da2c

