# Supplementary figures and images for: Dedicated Pediatricians in Emergency Department: Shorter Waiting Times and Lower Costs
Source: PLoS One. 2016 Aug 26;11(8):e0161149. doi: 10.1371/journal.pone.0161149 (PMC5001635; doi:10.1371/journal.pone.0161149)

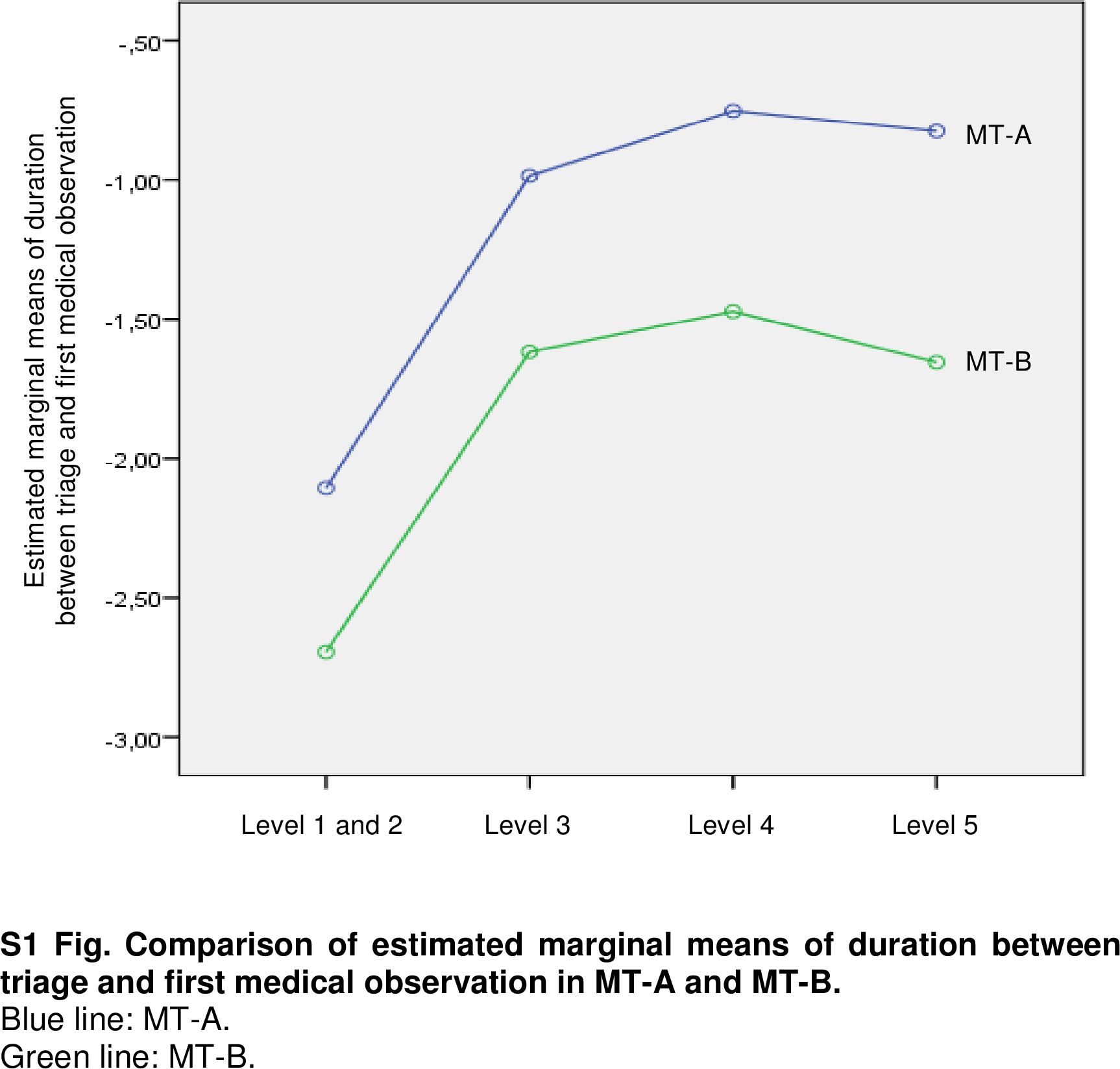

Supplement: S1 Fig — Blue line: MT-A. Green line: MT-B. (TIFF) [file pone.0161149.s001.tiff]

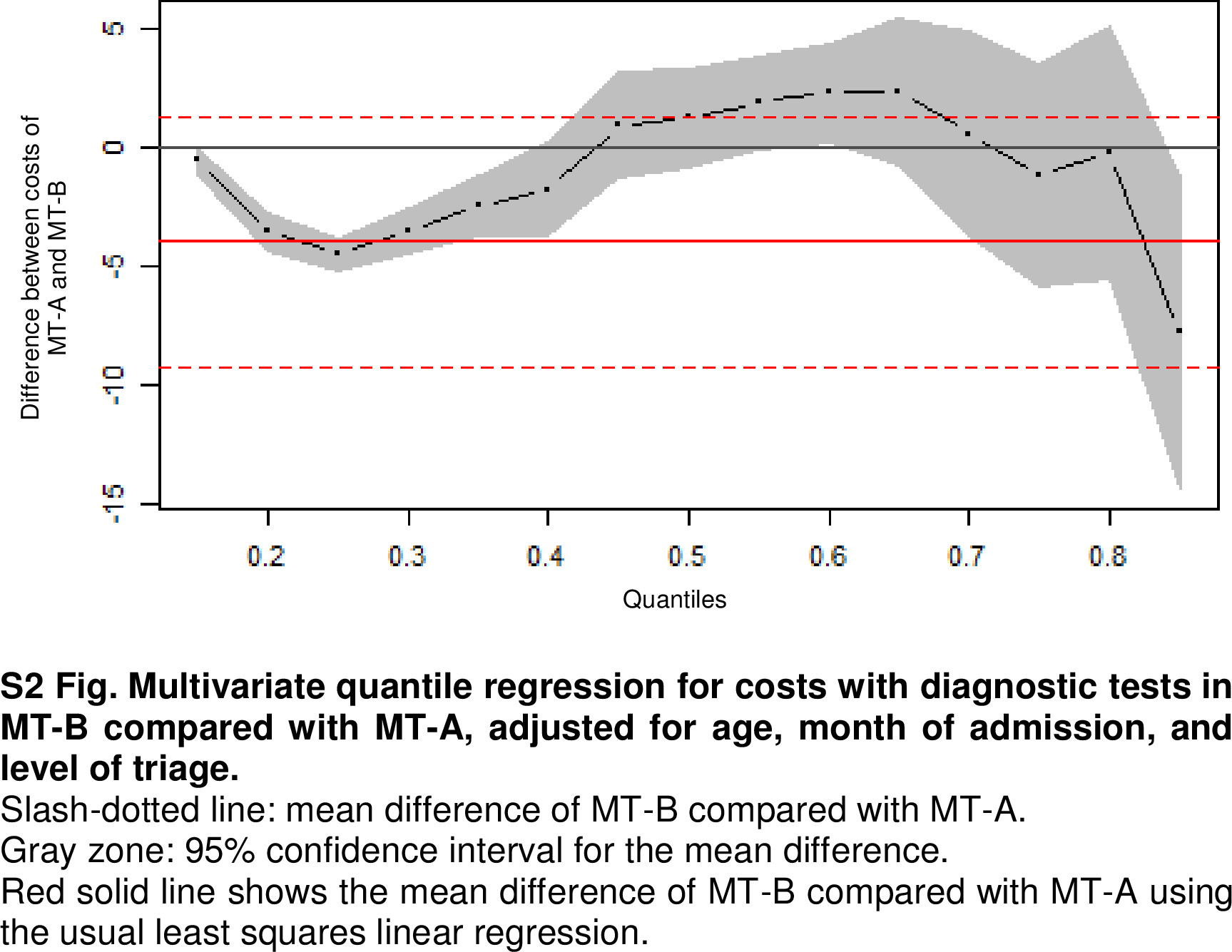

Supplement: S2 Fig — Slash-dotted line: mean difference of MT-B compared with MT-A. Gray zone: 95% confidence interval for the mean difference. Red solid line shows the mean difference of MT-B compared with MT-A using the usual least squares linear regression. (TIFF) [file pone.0161149.s002.tiff]
